# Association of acute kidney injury with frailty in elderly population: a systematic review and meta-analysis

**DOI:** 10.1080/0886022X.2019.1679644

**Published:** 2019-12-06

**Authors:** Zhu Liduzi Jiesisibieke, Tao-Hsin Tung, Qin-Yi Xu, Pei-En Chen, Shih-Yung Hsu, Yongguang Liu, Ching-Wen Chien

**Affiliations:** a Institute for Hospital Management, Tsing Hua University, Shenzhen Campus, Shenzhen, China;; b Hechi Third People’s Hospital, Guangxi, China;; c Department of Medical Research and Education, Cheng Hsin General Hospital, Taipei, Taiwan;; d Taiwan Association of Health Industry Management and Development, Taiwan;; e Department of Emergency Medicine, Cheng Hsin General Hospital, Taipei, Taiwan;; f Zhujiang Hospital, Southern Medical University Guangzhou, Guangzhou, China

**Keywords:** Acute kidney injury, elderly, frailty

## Abstract

**Aim:** The objective of this study was to assess whether an elderly patient’s frailty was associated with acute kidney injury (AKI) and to examine whether severe frailty group had an increased risk of AKI than mild–moderate group.

**Methods:** We searched The Cochrane Library, PubMed, and EMBASE for relevant studies without language limitations before 1 March 2019 with a priori defined inclusion and exclusion criteria. Five population-based cohort studies were included for systematic review and meta-analysis.

**Results:** Compared with the control group, the frailty group is significantly associated AKI (Odds Ratio = 2.05; 95% CI: 1.23–3.43). The moderate-severe frailty group has an increased risk of AKI than mild frailty group (Hazard Ratio = 2.87; 95% CI: 1.60–5.17.

**Conclusion:** In conclusion, the available best evidence support an association between frailty and AKI among elder patients, thus relevant interventions should be taken among elderly under potential risk of AKI.

## Introduction

Frailty is multidimensional syndrome defined as a ‘loss of physiologic reserve and the ability to resist stress’ [1,2], and frailty is common in geriatric populations and leads to a high risk of falls, disability, hospitalization and death [3]. The consequences of frailty are often measured mortality, morbidity and institutionalization [4]. Frailty also has been recognized as a risk factor for diabetes, cardiovascular, kidney dysfunction and depressed mood [5–7]. Frailty was thought to have physical and cognitive components [8]. Cognitive frailty may lead to impaired executive function, falls, balance disturbances functional decline, urge incontinence, disability [9]. Physical frailty severely limit patients’ ability to perform activities in home environment [6].

From the clinical viewpoint, acute kidney injury (AKI) is a complex clinical syndrome that significantly influences the disease process and worsens the outcome of a large number of patients in hospitalizations. Evidence-based studies defined the syndrome more accurately and elucidate the pathogenesis of AKI [10]. Previous epidemiologic studies also indicated that AKI in the elderly is increasingly common and that there is an age-dependent relationship between AKI and older age [11,12]. In addition, AKI is obvious that all clinical phenotypes of AKI could not fit into a single pathophysiologic pathway [10].

Whether frailty is associated AKI is an important clinical question warranting investigation. However, the impacts of frailty on AKI among elderly is still unclear. The objective of this systematic review and meta-analysis was to assess the risk of AKI in elder people with frailty and to examine the respective risk estimates in those with mild and moderate-severe frailty.

## Materials and methods

### Literature search

**Figure 1.**
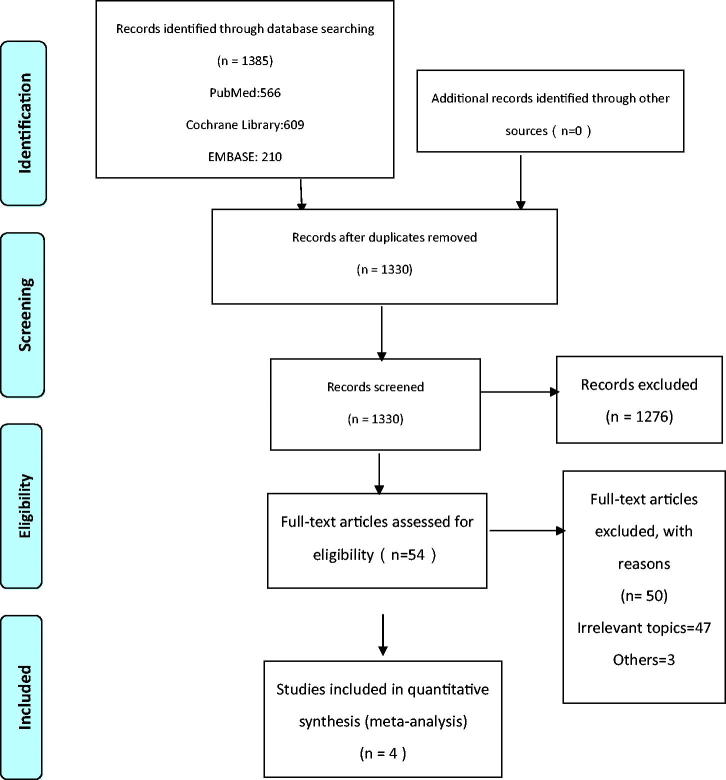
PRISMA study flow chart.

Electronic searches of The Cochrane Library, PubMed, and EMBASE for relevant studies from inception to 1st March 2019 were done in this study. The search string used was ‘(((elder people OR elderly OR the aged OR aging OR old people OR elder OR old man OR the old OR the elderly)) AND (frail OR frailty OR weak OR fragile OR feeble OR in poor health)) AND (acute renal injury OR acute kidney failure OR acute renal failure OR acute kidney injury OR acute kidney insufficiency OR acute renal insufficiency).’ with no limitations on language. We check the reference list of screening studies or other relevant comments to further identify other similar studies. The search strategy without handsearching is shown in Supplementary Material 1. This study is conducted according to the Preferred Reporting Items for Systematic Reviews and Meta-Analyses (PRISMA) guidelines (Figure 1). The protocol of this systematic review was registered in the PROSPERO under the number CRD136855.

### Study selection

These studies are included if the following inclusion criteria are met: 1. the study design was cohort study; 2. the exposure group was composed of people diagnosed with AKI and the control group was composed non-AKI; and 3. the outcome was either the odds or risk of AKI in subjects with frailty. The outcome of our interest was whether frailty existed before diagnosed with AKI. We did not include frailty after AKI. The titles and/or abstracts of the search results were scanned, and we obtained the full text when a study had appeared to meet the criteria mentioned before. In order to determine if there were potentially related information, we examined the full text. We screened the titles and abstracts of the search papers and obtained the full text when a study revealed to meet the inclusion criteria. We further examined the full text to decide whether information was potentially related. Two authors (Miss Zhu Liduzi Jiesisibieke and Dr. Tao-Hsin Tung) selected relevant studies independently, with disagreements resolved via discussion with a third senior author (Prof. Ching-Wen Chien).

### Data extraction and quality assessment

The following data were extracted from the included studies by using a data extraction form: first author, publication year, country, database used, study duration, study design, study subjects, mean age of study subjects, assigned groups and outcomes. The Newcastle–Ottawa Scale (NOS) were applied to assess the quality of the included studies. The NOS application includes three aspects: selection of study groups, comparability and outcome assessment, and they were used to assess the quality of the cohort studies [13]. For each item in the selection and result fields, a study of up to one star can be awarded, and for comparability, up to two stars can be obtained. If we get seven or more stars, we will consider conducting high quality research.

In addition, to enhance the reproducibility and comparability of this review to future reviews of a similar topic, we also included a risk of bias assessment using Risk of Bias in Nonrandomized studies of Interventions (ROBINS-I), since it is the newest and most robust method of assessing risk of bias in systematic reviews and meta-analyses.

### Inter-rater reliability for the selection and data extraction

To establish a consistent selection and data extraction, the kappa statistic was used to assess interobserver reliability between the two independent reviewers. Our study on interobserver reliability showed a kappa value of 0.798 for the data extraction (95% confidence interval (CI): 0.718–0.878).

### Statistical analysis

In this study, we used the Review Manager 5.3 (The Nordic Cochrane Center, The Cochrane Collaboration, 2014). We presented the risk of AKI as OR with 95% CI and assessed heterogeneity by using the I^2^ statistic. The I^2^ statistic evaluates the degree of variation across studies due to heterogeneity rather than by chance alone. An I^2^ values of 50% or more represents substantial heterogeneity [14]. Since I^2^ was below 50% (I^2^ = 43%), fixed-effect model were carried in meta-analysis.

## Results

### Characteristics of included studies

As illustrated in Figure 1, our search identified 1096 articles after removing duplicates. Eventually, 4 publications, reporting a total of four cohort studies with a total of 1052 study subjects met our inclusion criteria. The 2012 article by Aitken et al. reported one cohort study. The characteristics of the included studies are shown in Table 1. These studies were published between 2016 and 2018. All four cohort studies were population based cohort studies from the UK, the USA, and Korea. Furthermore, all selected studies were rated nine stars on the NOS scale considered with high quality and low-to-moderate risk using ROBINS-I. As shown in Tables 1 and 2, various sets of diagnostic codes were used for outcome measurement.

**Table 1.** Characteristics of included studies.

Number	Study, year, country, database used	Study Design	Study Duration	Study subjects	Mean age of study subjects	Assigned Groups	Outcomes	NOS Score^a^
1	Khaled Abdel-Kader, 2018, USA, Pubmed	Secondary analysis of a prospective cohort study	4 years (2007–2010)	243 AKI 74 No-AKI	57(AKI); 56(No-AKI)	According to Clinical Frailty Scale, patients are classified to 7 groups.	AKI was associated with higher CFS scores at 3 and 12 months.	S**** C** O***
2	Bellal Joseph, 2016, USA, Pubmed	Prospective cohort study	2 years (2013–2014)	93 Nonfrail; 139 Pre-frail; 136 Frail	68.52 ± 9.55(Non-frail); 74.71 ± 9.74(Pre-frail); 78.83 ± 10.63(Frail)	AKI and No-AKI	Frail patients were more likely to develop AKI. (P = 0.03） OR = 2.39, p = 0.004	S*** C** O***
3	Sarah Marton, 2018, UK, Pubmed	Prospective cohort study	2 weeks following admission	31 AKI 133 No-AKI	82.6 ± 7.5(AKI); 81.2 ± 8.3(No-AKI)	Severe frailty Mild-moderate frailty No frailty	Severe frailty was associated with AKI (p = 0.01).	S** C** O**
4	Seon Ha Beak, 2016, Korea, Pubmed	Retrospective cohort study	1 year (2013)	183 mild frail; 199 moderate frail; 151 severe frail	73.8 ± 4.7(mild frail); 76.5 ± 5.4(moderate frail); 79.0 ± 6.2(Severe frail)	AKI and Non-AKI	The frailest group had an increased risk of AKI than other groups.	S**** C** O***

^a^scale domains: S-selection of study group; C-comparability; O-outcome assessment

**Table 2.** Risk of bias assessment using ROBINS-I.

Author	Types of research	Pre-intervention		At intervention	Postintervention				Total
		Bias due to confounding	Bias in selection of participants into study	Bias in classification of interventions	Bias due to deviations from intended interventions	Bias due to missing data	Bias in measurement of outcomes	Bias in selection of the reported outcomes	Total bias
Khaled Abdel-Kader (2018)	Prospective cohort study	Low risk	Low risk	Low risk	Low risk	Low risk	Low risk	Low risk	Low risk
Bellal Joseph (2016)	Prospective cohort study	Low risk	Moderate risk	Low risk	Moderate risk	Low risk	Low risk	Low risk	Low risk
Sarah Marton (2018)	Prospective cohort study	Low risk	Moderate risk	Low risk	Low risk	Low risk	Moderate risk	Low risk	Moderate risk
Seon Ha Beak (2016)	Retrospective cohort study	Low risk	Low risk	Low risk	Low risk	Low risk	Low risk	Low risk	Low risk

In addition, Khaled-Adbel-Kader et al. [15], Seon Ha Baek et al. [16], and Sarah Morton et al. [17] staged AKI based on the criteria of Kidney Disease Improving Global Outcomes (KDIGO) by the difference between baseline and peak serum creatinine in the elder population. Bellal Joseph et al. [18] defined AKI as one of in-hospital complications. For the frailty, Khaled Abdel-Kader et al. [15] defined clinical frailty status using the Clinical Frailty Scale at 3 and 12 months after discharge. In Bellal Joseph et al. [18] study, frailty was measured by Trauma Specific Frailty Index (TSFI). Seon Ha Baek et al. [16] defined frailty based on the criteria of comprehensive geriatric assessment (CGA). Sarah Morton et al. [17] used ‘The Clinical Frailty Score’ (CFS) as a tool to assess elder patients’ frailty status.

### Association between frailty and AKI among the elderly population

**Figure 2.**
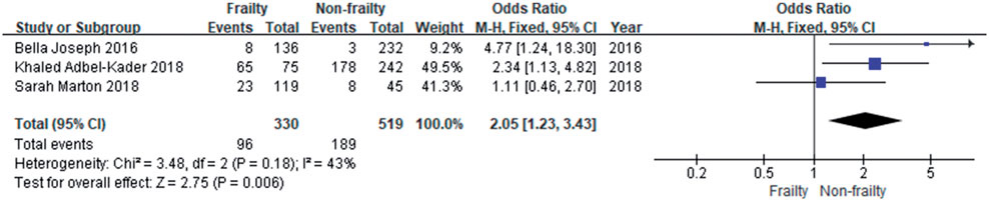
Odds of AKI in elder patients with frailty. CI: confidence interval; SE: standard error.

Four studies provided data on this outcome. The study by Joseph et al. [18] included 136 patients with frailty, 232 control subjects and concluded that frailty is a significant predictor of incident in-hospital complications, total mortality, and failure to rescue. The study by Abdel-Kader et al. included 242 patients with frailty and 75 control subjects. The results suggested that the main survivors of critical complications by AKI are clinically frail at three and twelve months after discharge, and AKI is independently related to frailty [15]. The study by Morton et al. included 45 patients with frailty and 119 control subjects. They showed that that the incident AKI in elderly acute medical patients is significantly related to severe frailty and higher mortality [17]. All these study provide odds ratio (OR) or risk ratio (RR), the total OR is 2.05 (95%CI: 1.23–3.43). The study by Atiken et al. include 248 frailty and 1577 control subjects. No meta-analysis for this outcome was undertaken as only this paper provide hazard ratio (HR = 3.4, 95%CI: 2.96–3.84, *p* < 0.01) [14]. In this study, Figure 2 shows that frailty was significantly associated with AKI than nonfrailty group (OR = 2.05, 95%CI: 1.23–3.34).

### Association between different level of frailty and AKI

**Figure 3.**
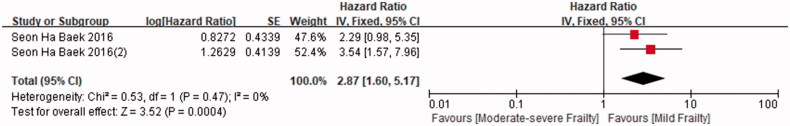
Risk of AKI in elder patients with frailty. CI: confidence interval; SE: standard error.

The aforementioned study by Marton et al. provided comparison among no frailty group, mild or moderate frailty, and severe frailty. The results showed that sever frailty group was significantly associated with AKI compared with mild–moderate frailty group (*p* = 0.011), while the other two groups (no-frailty vs. mild–moderate frailty; no-frailty vs. severe frailty) showed no significant difference [17]. Another study by Baek et al. included 54 frailty and 479 control subjects. This study showed that the frailest group had an increased risk of AKI than other two groups (HR = 3.54, *p* = 0.002) [16]. In this study, Figure 3 indicates that moderate–severe frailty was significantly related to AKI than mild-frailty group (HR = 2.87, 95%CI: 1.60–5.17).

## Discussion

### Clinical implications

To the best of our knowledge, this study is the first systematic review and meta-analysis to examine the impacts of frailty on AKI among the elderly population. Our study results support the hypothesis that elder patients with frailty had an increased risk of AKI, and as the degree of frailty deepens, the risk of AKI is increased. Thus, the overall evidence supported an association between frailty and AKI in the elder population.

AKI is common in hospital and is important for patients’ health [19]. Previous study estimated that the incidence of AKI is highest among elderly patients [12]. Frailty occurs most frequently in elderly population, and similar to AKI, carries high risk of adverse results include physical disability, decreased function, more hospitalization and higher mortality, especially in ill elderly patients critically admitted to the ICU [20]. Although the level of serum creatinine may not totally reflect patients’ kidney dysfunction in the elderly population, previous study found the J-shaped relationship between serum creatinine and limited ability and suggested that a lower serum creatinine may also be related to functional limitation and kidney function in this subgroup [21].

Some injury patterns of AKI, for example, inflammation and immune system dysregulation, may predispose to frailty. Other AKI consequences excluded fluid overload, anemia, cardiovascular disease, and metabolic derangements may also affect the frail status of some susceptible critical disease survivors [15,20]. Although frailty and AKI often occur in critically ill and elderly patients, the interactions between them remain unclear. Despite this, they may tend to cross each other in a vicious circle, thereby worsening the overall prognosis of the patients. To compare with younger patients, elder patients with AKI have decreased kidney function recovery and increased further mortality [22]. After adjustment for other confounding factors, old age is viewed as an independent predictor for AKI and a multifactorial disease process [23]. In addition, previous studies also demonstrated that both AKI and frailty were correlated to longer hospital stay or re-attendance rates [12]. Based on the results showed that no statistical difference in mortality between aged 65–80 years and more than 80 years, subjects’ frailty rather than biological age may be as a predictive factor for incident AKI [18]. Frailty is still the most problematic manifestation of population ageing [24]. Further studies are needed to validate the relationship, underpin potential mechanisms of possible bidirectional and develop treatment regimens focused on ameliorating the burden of frailty in elder survivors of critical illness and AKI.

To examine whether frailty had a significant impact on the rates of AKI among the old, our study included 4 cohort studies and excluded cross-sectional or case–control studies. Ordinary meta-analyses on the efficacy of interventions obtain high-quality evidence from randomized controlled trials only [13]. Randomized controlled trials plays an important role in health research because of its potential for control bias [25]; however, randomized trials are often not the best source of evidence on harm as the study duration is often too short to detect long-term or rare adverse outcomes [26,27]. In addition, it is not possible to randomly classify people into categories that are ‘Frail’ or ‘No Frailty’. All of our studies are cohort analysis is an advantage because these studies can detect the effects of long-term exposure to varying degrees of frailty. Our findings are important for identifying older patients who are more likely to have AKI. As result of that, medical staff could pay attention to nutrition, training and cognitive interventional approaches, which were proved to be effective to reverse frailty among the elderly patients [28].

### Methodological considerations

There are a few limitations in this study. Firstly, due to the amount of selected studies which could be search were not sufficient, the relative lower statistical power with smaller sample sizes is inevitable. Secondly, surrounding random-effects model is controversial, that is, the assumption of normal distribution for random effects against the principle of randomization in statistical inference [29]. If there were no random effects, the variance of random effects would be only as an encumbrance variable. The statistical application of this nuisance variable to meta-analysis weights would then be to markedly increase variance estimated and uniform the weights through grueling the larger studies [30]. Thirdly, it is difficult to conduct subgroup analyses based on demographic variables such as age, sex, and concurrent health status because the selected studies did not include adequate information. Future studies should be conducted to examine outcomes and confirm whether elderly patients’ frailty is an independent risk factor for AKI. Fourthly, I^2^ test seeks to determine whether there are real differences according to the results of the selected studies, namely, heterogeneity, or whether the variation in findings is reconcilable with chance alone, that is, homogeneity. I^2^ values of 0–24.9%, 25–49.9%, 50–74.9%, and 75–100% were viewed as none, low, moderate, and high heterogeneity, respectively. In this study, we used the fixed-effect model when I^2^ statistics was 43% less than 50%. However, we aggregate studies that are different methodologies, heterogeneity in the results is still inevitable. Fifthly, it is very difficult to explore the results of subgroup analysis for AKI and AKI requiring dialysis due to insufficient information for selected studies. Further studies are needed to estimate the pathological mechanisms associating frailty and AKI only or AKI with dialysis in elderly patients. Finally, although the kappa statistics for the agreement of interobserver reliability seemed acceptable [31], nondifferential misclassification or bias data extraction still may have occurred.

## Conclusion

In conclusion, our study suggests a relationship between frailty and AKI among elderly population. To further examine this finding and establish a stronger result, more large-scale prospective studies are warranted to provide more information about the details of the association between frailty and AKI. An increased rate of AKI occurs in frailty elderly patients and clinicians should be aware of this possibility.
